# Cerebral Edema Secondary to Heavy Metal Toxicity From Siddha Medicine: A Case Report and Case‐Based Review

**DOI:** 10.1155/crnm/9945172

**Published:** 2026-02-12

**Authors:** Zhibin Tan, Bernard Ji Guang Chua, Mingwei Ng, Sophia Reyes, Stella Setiawan, You Jiang Tan, Boon Kiat Kenneth Tan, Ponampalam R.

**Affiliations:** ^1^ Department of Neurology, National Neuroscience Institute, Singapore, nni.com.sg; ^2^ Neuroscience Academic Clinical Programme, SingHealth Duke-NUS Academic Medical Centre, Singapore; ^3^ Department of Oncology, National Cancer Centre Singapore, Singapore, nccs.com.sg; ^4^ Department of Emergency Medicine, Singapore General Hospital, Singapore, sgh.com.sg

**Keywords:** arsenic, cerebral edema, lead, mercury, poisoning, Siddha, toxicity

## Abstract

**Background:**

Traditional Indian medicine has been used to treat a variety of illnesses for centuries. However, there are existing concerns surrounding its heavy metal content. Amongst the different systems of traditional Indian medicine, Ayurvedic medicine has been most frequently implicated in heavy metal toxicity, while Siddha medicine has only very rarely been reported in association with lead and mercury poisoning.

**Case Presentation:**

A 58‐year‐old woman presented twice with severe headache, vomiting, dysphasia, truncal ataxia, and tremors. In the second presentation, she also had a clinical seizure. Both times, brain imaging showed diffuse gyral edema, but blood and cerebrospinal fluid tests were unyielding for any explanatory biochemical or serological causes. Both times, the patient displayed clinical and radiologic improvement during her inpatient stay, without any definitive treatment instituted. Eventually, a history of daily Siddha medicine usage for 3 months prior to presentation, as well as in the intervening period between both admissions, was elicited. Blood tests showed high lead levels beyond the assay’s limit for accurate quantification. Pharmaceutical analysis of her Siddha medicine capsules revealed contents of arsenic, mercury, and lead at levels that were several thousand times the safe limits. She was treated with oral dimercaptosuccinic acid with significant improvement in symptoms.

**Conclusion:**

We presented an unusual case of generalized cerebral edema caused by multiple metal toxicity from Siddha medicine use. Our case illustrates that Siddha medicine can cause a mixed toxidrome with toxicities from multiple metals at once. Our patient’s highly unusual presenting syndrome, including diffuse cerebral edema on brain imaging and triphasic waves on electroencephalogram, will hopefully be informative for physicians faced with similar cases in future.

## 1. Introduction

Traditional Indian medicine is one of the oldest medical practices in the world. Based on elemental and humoral natural philosophies of human physiology, it comprises systems of different cultural origins, including Ayurveda, Unani, and Siddha [1]. While there has been limited scientific evidence of their effectiveness, studies have suggested possible benefits in lowering blood glucose, the treatment of fistula‐in‐ano, and improving memory among children with mental retardation [2]. However, there are significant concerns amongst healthcare professionals regarding the safety of their use. Toxicity from heavy metals, for example, has been frequently reported in the use of Ayurvedic medications [3–5]. Heavy metal toxicity has been much less commonly associated with Siddha medicine, with only a few reports of lead and mercury poisoning [6–8].

Herein, we describe a case of multiple heavy metal toxicity (arsenic, mercury, and lead), with an uncommon presentation of diffuse cerebral edema from the use of traditional Indian remedies purporting to be Siddha medicine.

## 2. Case Presentation

**Figure 1:**
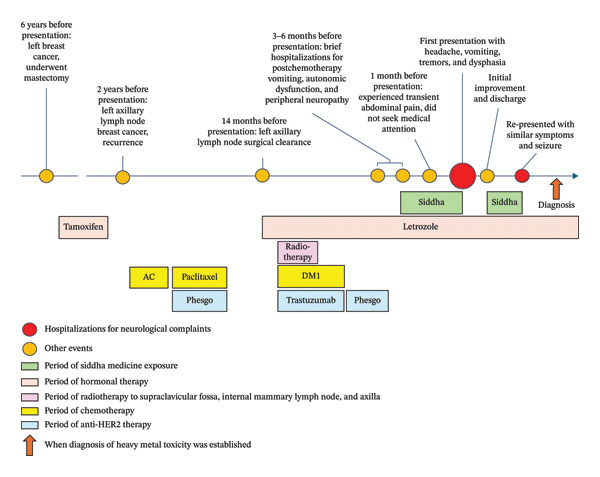
Timeline of events and medication exposure. Bisoprolol, though prescribed, is omitted from the figure, as it was not germane to the case presentation. AC: Adriamycin (doxorubicin) and cyclophosphamide; DM1: emtansine.

A 58‐year‐old Indian lady, who worked as an accountant, presented to the emergency department of a tertiary hospital in Singapore with a one‐month history of severe generalized headache, vomiting, postural tremors in all her limbs, and expressive dysphasia. She had a history of invasive left breast carcinoma (estrogen and progesterone receptors positive and c‐erb‐B2 negative), for which she had undergone a combination of neoadjuvant chemotherapy, surgical resection with lymph node clearance, and adjuvant chemo‐, radio‐, and anti‐HER2 therapy (Figure 1). She had completed her cancer treatment five months before and was only taking letrozole and bisoprolol at presentation. Computed tomography (CT) scans performed about two months prior to admission had not shown any evidence of residual cancer. Three to six months prior to presentation, she had brief hospitalizations for various chemotherapy complications, such as postchemotherapy vomiting, orthostatic hypotension due to autonomic dysfunction, and peripheral neuropathy. There was no antecedent febrile illness or vaccine administration. However, she reported transient abdominal pain lasting for several days, occurring about 1 month prior to presentation, which she could not characterize in greater detail.

On examination, she was alert (Glasgow Coma Scale 14) but disoriented and inattentive. Her verbal responses were slow, nonfluent, and palilalic. Fundoscopy was unremarkable. There were prominent bilateral, high‐frequency (9–11 Hz), low‐amplitude postural tremors in all limbs and her tendon stretch reflexes were diminished. She had no limb weakness, and sensory examination was unremarkable (including both pin‐prick sensation and proprioception) except for subjective non‐painful distal paresthesia in the lower limbs, which she said had been present since she received chemotherapy. She displayed truncal ataxia, causing an unsteady gait, which was compounded by symptomatic postural hypotension.

**Figure 2:**
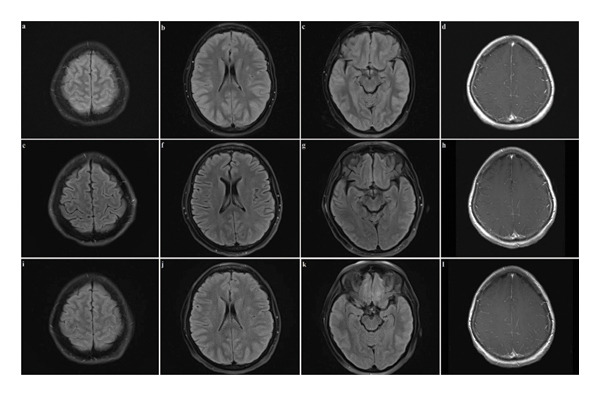
Magnetic resonance imaging (MRI) scan of the axial images of the brain. (a), (b), (c) T2 fluid‐attenuated inversion recovery (FLAIR) images acquired on Day 4 of initial presentation, showing diffuse gyral edema, sulcal and ventricular effacement, and sulcal hyperintensities. (d) Contrast‐enhanced T1 image acquired on Day 4, showing slightly increased sulcal enhancement. (e), (f), (g) T2 FLAIR images acquired on Day 19, showing resolution of the findings at Day 4. (h) Contrast‐enhanced T1 image acquired on Day 19, showing resolution of findings at Day 4. (i), (j), (k) T2 FLAIR images acquired upon repeat presentation, showing recurrence of the abnormalities found on Day 4 of initial presentation. (l) Contrast‐enhanced T1 image acquired upon repeat presentation, showing recurrence of mild sulcal enhancement.

**Figure 3:**
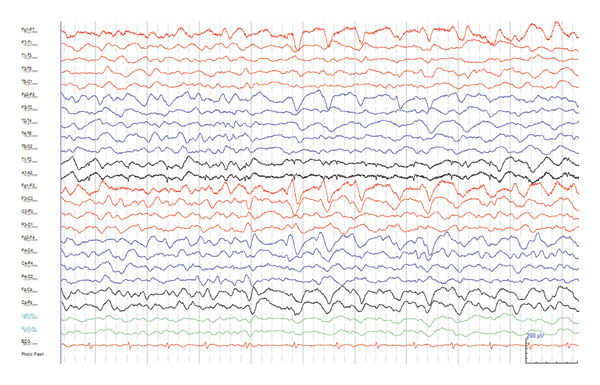
Scalp electroencephalogram performed during the first admission, showing triphasic waves on a background of generalized slow activity. Shown in bipolar montage, with sensitivity set at 10 μV/mm, low pass filter at 70 Hz, high pass filter at 0.5 Hz, and time base at 10 s per page. Channels are labeled according to the 10–20 system.

Biochemical, serological tests, and onconeural antibody tests were unremarkable (Supporting information 1). There was no leukopenia on complete blood count. Blood and urine drug screening tests were likewise unyielding. Magnetic resonance imaging (MRI) scan of the brain performed on Day 4 of presentation showed diffuse gyral edema and mild post‐gadolinium sulcal enhancement (Figure 2). A CT venogram was performed, returning negative for cerebral venous thrombosis. Electroencephalogram (EEG) showed generalized triphasic waves and continuous slow activity (Figure 3).

The patient underwent a lumbar puncture on Day 10 of presentation, but cerebrospinal fluid (CSF) analysis results were unremarkable except for elevated CSF protein (Supporting Information 1). No malignant cells were identified on CSF cytology. Amidst these extensive investigations, and despite receiving no specific treatment, the patient displayed a gradual but significant recovery over the succeeding 2 weeks. A repeat MRI brain scan showed resolution of diffuse cerebral edema (Figure 2). The patient was discharged on Day 20 of presentation, by which time her dysphasia and nausea had resolved, though she continued to experience residual postural tremors and mild episodic headaches.

Five weeks after discharge, the patient was readmitted with a recurrence of the same symptoms. On Day 3 of this second admission, she had a witnessed, self‐terminating, bilateral, myoclonic seizure with impaired consciousness. She was hence commenced on levetiracetam.

She underwent yet another MRI brain scan, showing a recurrence of diffuse cerebral edema. A repeat lumbar puncture was, once again, unyielding except for raised CSF protein, and CSF cytology again showed no malignant cells. Like her first presentation, her symptoms gradually improved without any treatment; her headaches and nausea resolved while her mentation and tremors improved.

**Figure 4:**
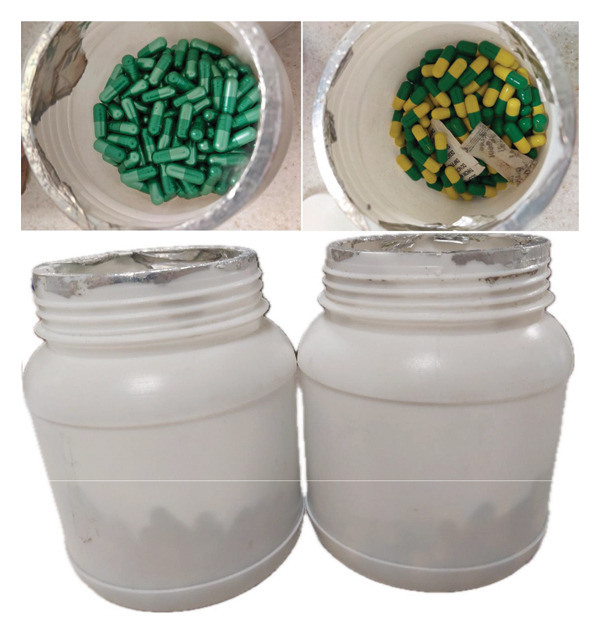
Capsules of Siddha medicine that the patient had been taking. Two of the green/dark green capsules were taken every morning, and two of the green/yellow capsules were taken every evening.

Later during the admission, it became known that the patient had been taking daily doses of an unlabeled Siddha medication (Figure 4) for its purported anti‐cancer effects, for 3 months before her first presentation, as well as during the intervening period between her two admissions.

Acting on a suspicion of heavy metal toxicity, the patient’s blood samples were drawn on Day 33 of her second admission for testing. Her blood lead concentration was found to be more than five times the upper limit of normal (Table 1).

**Table 1:** Results of blood tests for metals.

Metal tested	Blood concentration (normal laboratory reference range in parentheses)
Copper	1063 μg/L (734–1714)
Arsenic	5.6 μg/L (< 31)
Cadmium	< 1.0 μg/L (< 2.7)
Mercury	5.6 μg/L (< 22)
Lead	993 μg/L (< 100)

The Siddha medicine capsules were sent for pharmaceutical analysis and were found to contain very high concentrations of arsenic (up to 816‐fold above the permissible level), mercury (up to 75,966‐fold the permissible level), and lead (up to 2,215‐fold the permissible level). The details of the metal content of these capsules, in parts per million, are shown in Table 2.

**Table 2:** Results of metal analysis of Siddha medicine capsules by inductively coupled plasma mass spectrometry.

Metal tested	Heavy metal analysis of green/dark green capsules (ppm)	Heavy metal analysis of green/yellow capsules (ppm)	HSA permissible levels (ppm)
Copper	Not detected	Not detected	—
Arsenic	3226.70	4078.85	≤ 5
Cadmium	Not detected	Not detected	—
Mercury	37982.77	5064.38	≤ 0.5
Lead	22145.57	13756.32	≤ 10

*Note:* HSA, Health Sciences Authority of Singapore.

Abbreviation: ppm, parts per million.

The patient discontinued her use of Siddha medication and was treated with a 3‐week course of oral dimercaptosuccinic acid (DMSA) for chelation of metals. Serum lead levels initially improved to 461 μg/L 1‐week post‐chelation therapy but rose again to 582 μg/L after another 4 weeks despite the patient having no significant repeat exposure to exogenous lead. She was given another 3‐week course of DMSA, after which the lead level improved to 367 μg/L three weeks post‐chelation. An MRI scan of the brain was repeated 18 weeks after her first presentation to us (two weeks post‐chelation therapy, and 10 weeks after the last MRI scan), demonstrating a resolution of all described abnormalities. Anemia, which was previously thought to be due to chemotherapy, improved from a hemoglobin level of 7.3 to 12.0 g/dL after chelation therapy. She was reviewed after completion of her chelation therapy (5 months since last Siddha medicine exposure) and reported significant improvement in speech, postural dizziness, and tremors. There had been no recurrence of confusion, truncal ataxia, or abdominal pain.

## 3. Discussion

Siddha medicine originated from South India, during the Sangam era of Indian history, specifically from a class of Tamil sages (i.e., Siddhas) who were believed to be “perfect” or “immortal” [9]. This ostensible immortality of the Siddhas was attributed to alchemical practices and the use of elixirs containing metals thought to confer a host of physical and spiritual benefits, in synergy with herbs [10]. Therefore, the preparation of Siddha drugs involves the deliberate processing of metals (including mercury, mica, copper, and arsenic, among others) together with certain combinations of herbs [10]. These metals are mined from ores at auspicious times, purified by heat in earthen crucibles (calcination), repeatedly boiled or soaked in decoctions such as plant juices and cow’s urine (detoxification), and ground with herbal pastes [11, 12].

Several important lessons may be derived from our case of heavy metal toxicity from Siddha medicine use.

First, Siddha medicine may be associated with heavy metal toxicity. Although heavy metal toxicity has been associated with traditional Indian remedies, it is mostly Ayurvedic preparations that have been reported to contain unsafe levels of heavy metals [3–5]. Siddha medicine has been less commonly implicated; in fact, several studies have reported safe levels of heavy metal content in Siddha formulations [13–15]. To the best of our knowledge, there have only been three reports of heavy metal toxicity resulting from Siddha medicine use. Janardhanan et al. described one case of lead poisoning after a 10‐day course of Siddha medicine for the treatment of diabetes, presenting with intractable abdominal pain, and who was successfully treated with intramuscular dimercaprol [6]. Doshi et al. described five cases of nephrotic syndrome from chronic mercury poisoning; oral chelation with dimercaptopropane‐1‐sulfonic acid led to clinical improvement and successful elimination of the metal [7]. Gnanashanmugam et al. reported mercury toxicity in 32 patients who had presented with a range of neuromyotonic symptoms, e.g., dysesthesias, myokymia, fasciculations, autonomic dysfunction, and encephalopathy, following ingestion of Siddha medicines [8]. We hope that our case can add to the scarce literature on Siddha medicine causing heavy metal toxicity and raise awareness among physicians so that metal toxicity can be appropriately considered among the differential diagnoses in the appropriate context.

Second, Siddha medicine can contain unsafe levels of multiple metals at the same time, hence causing a mixed toxidrome. While our patient had been exposed to unsafe levels of three different metals (arsenic, mercury, and lead), only lead was detected in her blood samples dispatched 33 days after her last exposure to the culprit Siddha medicine capsules. Arsenic and mercury were not found in high concentrations in her blood, likely because of the relatively short half‐lives of arsenic (up to 2 days) and mercury (24–40 h when ingested as inorganic mercury) [16–19]. In contrast, lead has a much longer half‐life of 1‐2 months in the blood and was hence readily detected beyond the detection limit despite the late blood draw [20]. Therefore, considering the late blood draw, the metal(s) responsible for her presenting syndrome may have been any combination of the arsenic, mercury, and lead found to be present at unsafe levels on analysis of her Siddha medicine capsules. It is also noteworthy that many of our patient’s manifestations had resolved or improved (headache, nausea, mentation, and tremors) despite her blood lead level remaining several times the upper limit of normal, hence suggesting that some of these manifestations may have been due to transiently high blood levels of arsenic or mercury that were undocumented. Our case highlights the need to consider the timing of blood or tissue sampling, the half‐lives of the toxins being tested for, and analysis of the suspected source for accurate diagnosis. Table 3 shows the typical sources, symptoms, and signs of toxicity due to each metal. A detailed analysis of our patient’s clinical manifestations, by the possible culprit metal, can be found in Table 4.

**Table 3:** Typical sources, symptoms, and signs of toxicity of arsenic, mercury, and lead.

Metal	Typical sources of exposure	Symptoms and signs	Chelating agent options [21–23]
Arsenic	In general [19]: • Elemental arsenic is nontoxic • Organic arsenicals (e.g., arsenobetaine) pose a low risk of toxicity • Inorganic arsenicals are the primary cause of arsenic toxicity Contaminated drinking water (e.g., groundwater from wells) [24] Contaminated soil, agricultural products, and seafood [19, 25] Occupational (production of pesticides and herbicides, smelting, mining, glass manufacturing, semiconductor manufacturing, and carpentry involving arsenate wood preservatives) [25]	Acute intoxication [19, 26]: • Nausea, vomiting, colicky abdominal pain, and profuse watery or bloody diarrhea • Hypotension, heart failure, arrhythmias (e.g., QTc prolongation or ventricular arrhythmias), pericardial effusion, and myocarditis • Pulmonary edema and respiratory failure • Fluid and electrolyte disturbances (hypokalemia and hypomagnesemia) • Electrocardiogram abnormalities • Mental status changes Subacute exposure (onset 2–8 weeks) [19]: • Peripheral neuropathy (symmetric sensorimotor neuropathy, which may mimic Guillain–Barré syndrome, axonal on electrophysiological studies, can be painful) Chronic exposure [19, 25]: • Neurobehavioral changes (can be exacerbated by lead exposure) including deficient verbal intelligence and long‐term memory • Peripheral neuropathy • Increased fetal mortality and preterm birth • Macrocytosis and pancytopenia • Hyperkeratotic lesions on extremities, melanosis, bronze pigmentation of skin, and Mee’s lines on fingernails • Liver disease • Metallic taste • Carcinogenic effects, especially associated with cancers of the skin, lungs, and bladder	• DMSA (limited CNS penetration) • DMPS (limited CNS penetration) • BAL (some CNS penetration) • Penicillamine (poor CNS penetration)

Mercury	Elemental mercury can be inhaled, and organic and inorganic mercury are most commonly ingested [19] Occupational (mercury or gold mining and industries making antiseptics, cosmetics, explosives, dyes, and pigments), food (grain contaminated by fungicide or fish from contaminated water bodies) [19, 27], and thimerosal in certain vaccines [19]	When inhaled as elemental mercury [19]: • Chills • Gastrointestinal upset and poor appetite • Renal failure • Cough, dyspnea, and adult respiratory distress syndrome • Acrodynia, pruritus, oral ulcers, and loose teeth • Weakness, myalgia, and dysautonomia • Headache • Erethism (memory loss, drowsiness, lethargy, depression, withdrawal, and irritability) • Dry mouth When ingested as inorganic mercury [19, 27]: • Nausea, vomiting, abdominal pain, and hemorrhagic gastroenteritis • Acute tubular necrosis • Hypotension • Membranous glomerulonephritis and nephrotic syndrome • Acrodynia • Erethism When exposed dermally as inorganic mercury [19]: • Hyperpigmentation and swelling or skin, vesicular or scaly rash • Loosening of teeth and bluish discoloration of the gingiva When ingested as organic mercury [19]: • Concentric construction of bilateral visual fields • Paresthesia of extremities and mouth and sensory peripheral neuropathy • Incoordination, ataxia, tremors, chorea, and Minamata disease • Mental retardation • Dysarthria and hypersalivation • Auditory impairment • Damage to gray matter of cerebral and cerebellar cortex (especially calcarine region, occipital lobe, and pre‐ and postcentral temporal cortex)	Elemental/inhaled mercury chelators: • DMSA or DMPS initially • Acetylcysteine for continued chelation Organic mercury chelators: • Acetylcysteine (penetrates CNS) • DMSA and DMPS not effective due to suboptimal CNS penetration causing inability to reach demethylated and dealkylated mercury Inorganic mercury chelators: • DMSA or DMPS only work if administered early (within a few days of exposure) but have limited effect for chronic exposure due to suboptimal CNS penetration • Acetylcysteine for continued chelation

Lead	Occupational (lead trade workers such as smelters, painters, and body and fender repairmen), leaded dishware, bootlegged moonshine liquor, cosmetics, and folk remedies [28]	No specific toxidrome, may present with a combination of any of the following: • Intractable colicky abdominal pain, constipation, hepatotoxicity, and pancreatitis [19, 28] • Motor clumsiness and peripheral neuropathy with weakness paralysis, including wrist or foot drop [28] • Coma, generalized encephalopathy, seizures, and ataxia (these tend to predominate with organic lead such as tetraethyl lead) [19] • Papilledema [19] • Cognitive impairment, personality changes, insomnia, irritability, hyperactivity, and developmental delay [19, 29] • Infertility, stillbirths, and neonatal deaths [28, 30] • Hypertension, renal failure, Fanconi syndrome, gout, immune imbalances, abnormalities of skeletal and dental abnormalities, and vitamin D deficiency [19, 28, 30] • Microcytic anemia [19] • Spontaneous abortion and premature birth [19] • Carcinogenic effects uncertain [28]	• DMSA • BAL • Penicillamine • Sodium calcium edetate

*Note:* BAL, British anti‐Lewisite (also known as dimercaprol); DMPS, 2,3‐dimercapto‐1‐propanesulfonic acid (also known as unithiol); DMSA, dimercaptosuccinic acid.

Abbreviation: CNS, central nervous system.

**Table 4:** Clinical manifestations of our patient and their possible culprit causes.

Clinical manifestation	Possible culprits
Transient abdominal pain prior to presentation	Arsenic, mercury, lead
Headache	Nonspecific, could be a symptom of generalized cerebral edema
Nausea and vomiting	Arsenic, mercury
Postural tremors	Mercury
Truncal ataxia	Mercury, lead
Speech disturbances	Arsenic, mercury
Altered mental status	Arsenic, mercury, lead
Orthostatic hypotension and autonomic dysfunction	Known issue attributed to chemotherapy prior to Siddha medicine exposure, but can be exacerbated by mercury
Nonpainful sensory‐predominant peripheral neuropathy	Known issue attributed to chemotherapy prior to Siddha medicine exposure, but can be exacerbated by arsenic, mercury, or lead
Anemia	Lead
Generalized cerebral edema	Mercury, lead

Next, our case demonstrated that metal toxicity can present with diffuse cerebral edema, which was a highly unusual presentation, making the diagnosis challenging to establish. Although an evaluation for infective, inflammatory, and immune‐mediated causes was performed, diffuse cerebral edema that resolved radiologically without any specific treatment was not consistent with any syndrome that we were aware of. The finding of triphasic waves on EEG is known to be a nonspecific sign that can be associated with a wide array of toxic‐metabolic encephalopathies, but it was not particularly helpful in our diagnostic evaluation, because triphasic waves can also be associated with non‐metabolic encephalopathies [31]. Furthermore, triphasic waves have never been reported in association with generalized cerebral edema due to heavy metal toxicity. Out of the three metals found in our patient’s capsules, lead (especially in organic form) is most strongly associated with a generalized encephalopathy with cerebral edema at toxic levels, though such a manifestation would be more likely in pediatric patients compared to adult patients [19, 32] Chronic exposure to organic mercury can also cause cerebral edema, as described in Minamata disease [19, 33].

Finally, we observed that our patient was having a temporary rise in serum lead level after the first course of chelation therapy, which is likely due to mobilization from skeletal stores. Bone tissue is known to be the chief target for lead in chronic exposure, and mobilization has been known to cause elevated lead levels, without exposure to exogenous sources [34, 35]. This is important information for clinicians treating patients with lead toxicity, since ignorance may spark an unnecessary hunt for additional sources of toxicity.

The main limitation of our case report is the late blood draw for heavy metal analysis, limiting our ability to draw clearer correlations between specific metals and our patient’s clinical manifestations. Nonetheless, the analysis results of our patient’s Siddha medicine capsules allowed us to at least identify three metals, each of which likely contributed to her toxidrome.

Another potential limitation was the lack of hair analysis. Hair analysis has been proposed as an option in the evaluation of suspected metal toxicity, especially after chronic exposure. In our patient, hair analysis may have been a useful adjunctive test, especially for arsenic and mercury, which we were unable to demonstrate via blood tests. However, hair analysis is fraught with several pitfalls, such as poorly established reference ranges, lack of validated analytic techniques, low inter‐laboratory reliability, and confounding by exogenous hair contaminants [36]. Therefore, we believe that hair analysis may not have been of much value in our patient’s case, given that we have already demonstrated extremely high levels of metal exposure via capsule analysis.

## 4. Conclusions

We presented an unusual case of generalized cerebral edema, with a hitherto little‐known cause of multiple metal toxicity, Siddha medicine. The lessons from our case, (1) that the use of Siddha medicine may be associated with metal toxicity, (2) the presence of multiple metals at unsafe levels can cause a mixed toxidrome that may require analysis of the source to better understand it, (3) metal toxicity can present radiologically with diffuse cerebral edema and electrographically with triphasic waves on scalp EEG, and (4) serum lead levels can rise temporarily after completion of chelation therapy due to mobilization of bone stores after chronic exposure, will hopefully be informative for clinicians faced with similar cases.

**Nomenclature**
CSF: Cerebrospinal fluid
CT: Computed tomography
EEG: Electroencephalogram
FLAIR: Fluid‐attenuated inversion recovery
HSA: Health Sciences Authority of Singapore
IRB: Institutional review board
MRI: Magnetic resonance imaging
ppm: Parts per million

## Author Contributions

Zhibin Tan, Bernard Ji Guang Chua, Mingwei Ng, Sophia Reyes, and Stella Setiawan were involved in conceptualization, data curation, investigation, project administration, validation, visualization, initial writing, reviewing, and editing. You Jiang Tan, Boon Kiat Kenneth Tan, and Ponampalam R. were involved in supervision, validation, reviewing, and editing.

## Funding

The authors received no specific funding for this work.

## Ethics Statement

As per our institution’s policy, institutional review board (IRB) approval is not required for case reports.

## Consent

The patient described in our case report has provided written consent for the publication of this case report. The data and pictures have been anonymized whenever possible.

## Conflicts of Interest

The authors declare no conflicts of interest.

## Supporting Information

Supplemental information 1: Table of unyielding blood and cerebrospinal fluid tests.

## Supporting information


**Supporting Information** Additional supporting information can be found online in the Supporting Information section.

## Data Availability

The data that support the findings of this study are available from the corresponding author upon reasonable request. The data are not publicly available due to privacy or ethical restrictions.
