# Supplementary material for: Cerebral Edema Secondary to Heavy Metal Toxicity From Siddha Medicine: A Case Report and Case‐Based Review
Source: Case Rep Neurol Med. 2026 Feb 12;2026:9945172. doi: 10.1155/crnm/9945172 (PMC12902456; doi:10.1155/crnm/9945172)
Supplement: Supplementary file 1 — Supporting Information Additional supporting information can be found online in the Supporting Information section. [file CRNM-2026-9945172-s001.docx]

**Supplemental information 1**. Table of unyielding blood and cerebrospinal fluid tests. AFB, acid-fast bacilli; AMPAR, alpha-amino-3-hydroxy-5-methyl-4-isoxazolepropionic acid receptors; CASPR2, contactin-associated protein-like 2; CMV, cytomegalovirus; CRMP5, Collapsin response-mediator protein 5; CSF, cerebrospinal fluid; DPPX, dipeptidyl-peptidase-like protein 6; GABA_B_R, Gamma aminobutyric acid receptor type B; GAD65, glutamic acid decarboxylase 65; HSV, herpes simplex virus; LGI1, Leucine-rich glioma-inactivated protein 1; MOG, myelin oligodendrocyte glycoprotein; NMDAR, N-methyl-D-aspartate receptor; PCR; polymerase chain reaction; SOX1, SRY box 1; ULN; upper limit of normal.

| **Blood hematological and biochemical tests** | **Result** |
| --- | --- |
| Hemoglobin | 7.3 g/dL (baseline 8-9) |
| Mean corpuscular volume | 78.2 fL |
| White blood cell count | 4.56 x 10^9^/L |
| Platelets | 172 x 10^9^/L |
| Peripheral blood film | Normocytes and microcytes with few macrocytes and tear-drop poikilocytes, slight anisocytosis, few elliptocytes |
| Urea, creatinine, and electrolytes | Normal |
| Liver enzymes | Normal |
| Thyroid stimulating hormone | Normal |
| Free thyroxine (T4) | 15.5 pmol/L (ULN 14.4) |
| Blood toxicology (acidic and neutral drugs panel) | Not detected |
| Blood toxicology (basic drugs panel) | Not detected |
| Blood toxicology (benzodiazepines, hypnotics, and opioids | Not detected |
|  |  |
| **Blood microbiological tests** |  |
| Blood aerobic and anaerobic cultures | Negative |
| Blood treponema pallidum antibody (TPPA) | Non-reactive |
|  |  |
| **Blood serological tests** |  |
| Antinuclear antibody (ANA) | Negative |
| Anti-double stranded DNA antibody | Negative |
| Anti-extractable nuclear antigen (ENA) antibodies (Smith, ribonucleoprotein, Ro, La, Scl 70, Jo-1) | Negative |
| Anti-neutrophil cytoplasmic antibody (ANCA) | Negative |
| Anti-myeloperoxidase (MPO) antibody | Negative |
| Anti-proteinase 3 (PR3) | Negative |
| Thyroid peroxidase (TPO) antibodies | Negative |
| Thyroglobulin antibodies | Negative |
| Anti-MOG IgG | Negative |
| Autoimmune encephalitis panel (NMDAR, CASPR2, AMPAR 1/2, LGI1, DPPX, GABA_B_R) | Negative |
| Paraneoplastic / onconeural panel (amphyphysin, CRMP5, Ta, Ri, Yo, Hu, recoverin, SOX1, titin, zic4, GAD65, Tr) | Negative |
|  |  |
| **Urine tests** |  |
| Urine toxicology (acidic and neutral drugs panel) | Not detected |
| Urine toxicology (basic drugs panel) | Bisoprolol detected |
| Urine toxicology (benzodiazepines, hypnotics, and opioids | Not detected |
|  |  |
| **CSF tests (first lumbar puncture)** |  |
| Opening pressure | 15.5 cmH_2_O |
| White blood cell count | 2 /µL |
| Differential | Few lymphocytes and monocytes |
| Red blood cell count | 0 /µL |
| Glucose | 3.7 mmol/L |
| Protein | 0.88 g/L (ULN 0.4) |
| Flow cytometry | Paucicellular sample without lymphocytes for analysis. |
| CSF microbiological tests, including FilmArray meningitis PCR panel, tetraplex PCR (HSV, CMV, VZV, toxoplasma), AFB smear, AFB culture, tuberculosis PCR, fungal culture, *Cryptococcus* antigen | Negative |
| Cytology | Rare atypical but degenerate cells |
| Autoimmune encephalitis panel (NMDAR, CASPR2, AMPA1/2, LGI1, DPPX, GABA_B_R) | Negative |
|  |  |
| **CSF tests (second lumbar puncture)** |  |
| White blood cell count | 2 /µL |
| Red blood cell count | 0 /µL |
| Glucose | 3.5 mmol/L |
| Protein | 0.53 g/L (ULN 0.4) |
| Flow cytometry | No CD19+ B cells in sample. There are ~ 33% CD3+ CD5+ T cells with normal CD4:CD8 ratio. |
| CSF microbiological tests, including AFB smear, AFB culture, tuberculosis PCR | Negative |
| Cytology | No malignant cells seen |
